# Dynamic structure of motor cortical neuron coactivity carries behaviorally relevant information

**DOI:** 10.1162/netn_a_00298

**Published:** 2023-06-30

**Authors:** Marina Sundiang, Nicholas G. Hatsopoulos, Jason N. MacLean

**Affiliations:** Committee on Computational Neuroscience, University of Chicago, Chicago, IL, USA; University of Chicago Neuroscience Institute, Chicago, IL, USA; Department of Organismal Biology and Anatomy, University of Chicago, Chicago, IL, USA; Department of Neurobiology, University of Chicago, Chicago, IL, USA

**Keywords:** Functional network, Motor cortex, Temporal network

## Abstract

Skillful, voluntary movements are underpinned by computations performed by networks of interconnected neurons in the primary motor cortex (M1). Computations are reflected by patterns of coactivity between neurons. Using pairwise spike time statistics, coactivity can be summarized as a *functional network* (*FN*). Here, we show that the structure of FNs constructed from an instructed-delay reach task in nonhuman primates is behaviorally specific: Low-dimensional embedding and graph alignment scores show that FNs constructed from closer target reach directions are also closer in network space. Using short intervals across a trial, we constructed *temporal FNs* and found that temporal FNs traverse a low-dimensional subspace in a reach-specific trajectory. Alignment scores show that FNs become separable and correspondingly decodable shortly after the *Instruction* cue. Finally, we observe that reciprocal connections in FNs transiently decrease following the *Instruction* cue, consistent with the hypothesis that information external to the recorded population temporarily alters the structure of the network at this moment.

## Author Summary

It remains unclear how motor cortical neurons flexibly perform the computations necessary to generate movement. We hypothesized that neuronal coactivity contains movement information, and its dynamics can reveal how the population switches computations during a task. We quantified coactivity as a functional network (FN) with single neurons as nodes and population coactivity as directed weighted edges. We also constructed FNs within short epochs across a trial to determine *when* coactivity begins to carry information and to investigate the dynamic structure of these interactions. Following the *Instruction* cue, reciprocal connections in FNs transiently decrease, and shortly after, FNs become maximally decodable for reach direction.

## INTRODUCTION

Individual neurons do not function in isolation, but rather as coactive and cooperatively interacting components of spiking networks. The resulting coordinated neuronal coactivity is reflected in pairwise spike time statistics. Spike time correlations have been implicated in the propagation of spiking in networks (Bojanek et al., 2020; Chambers & MacLean, 2016) making them an intriguing signal when studying the circuit mechanisms and computations that give rise to behavior. Recent work has shown that spike time correlations provide information, in addition to that provided by changes in firing rate, as to the nature of visual stimuli (Dechery & MacLean, 2018; Kotekal & MacLean, 2020; M. Levy et al., 2020; Ruda et al., 2020), auditory stimuli (Betzel et al., 2019; Insanally et al., 2019), and spatial location (E. R. J. Levy et al., 2021; Nardin et al., 2021). In motor cortex (M1) pairwise spike *count* correlations have similarly been shown to provide information about motor behavior beyond what is provided by firing rates alone (Maynard et al., 1999), and have also been used to improve encoding models that predict the activity of neurons (Stevenson et al., 2012). However, it remains unclear whether pairwise spike *time* correlations contain behaviorally relevant information. The temporal dynamics of spike time correlations over the course of a behavioral trial and the extent of specificity of spike time correlations to kinematic variables are consistently underexplored.

Here, we represent pairwise spike time statistics as *functional networks* (*FNs*) and use tools from network science (Bassett & Sporns, 2017; Bullmore & Sporns, 2009; Rubinov & Sporns, 2010) to examine the structure and information content of pairwise spike time correlated activity of active populations in M1. Given that many of the computations needed to carry out a movement happen in fractions of a second, averaging interactions over long periods of time is unlikely to capture important dynamic aspects of the network (Pedreschi et al., 2020). We extend the FN framework by constructing temporal FNs (Holme & Saramäki, 2012; Ju & Bassett, 2020; Thompson et al., 2017) of single trials by computing pairwise spike time statistics across short intervals to elucidate the temporal progression of pairwise correlations among a population of M1 neurons throughout a trial and to relate single-trial population dynamics to network structure.

Using temporal FNs, we evaluate whether the dynamics of pairwise correlations carry information about the instructed reach, and determine when network structure begins to carry information relative to behaviorally relevant moments during the trial. We also determine what types of network interactions are most prevalent relative to different phases of the trial. We find that temporal FNs evolve during the trial in a way that specifies the instructed reach. Using a perceptron decoder, we demonstrate that temporal FNs contain information about motor behavior beyond what is conveyed by firing rate changes alone. We show that the topology of temporal FNs varies systematically over the time course of a trial, becoming transiently less reciprocal after the onset of the *Instruction* cue, coinciding with the increase in decodable behavioral information from the FNs.

## RESULTS

**Figure 1.**
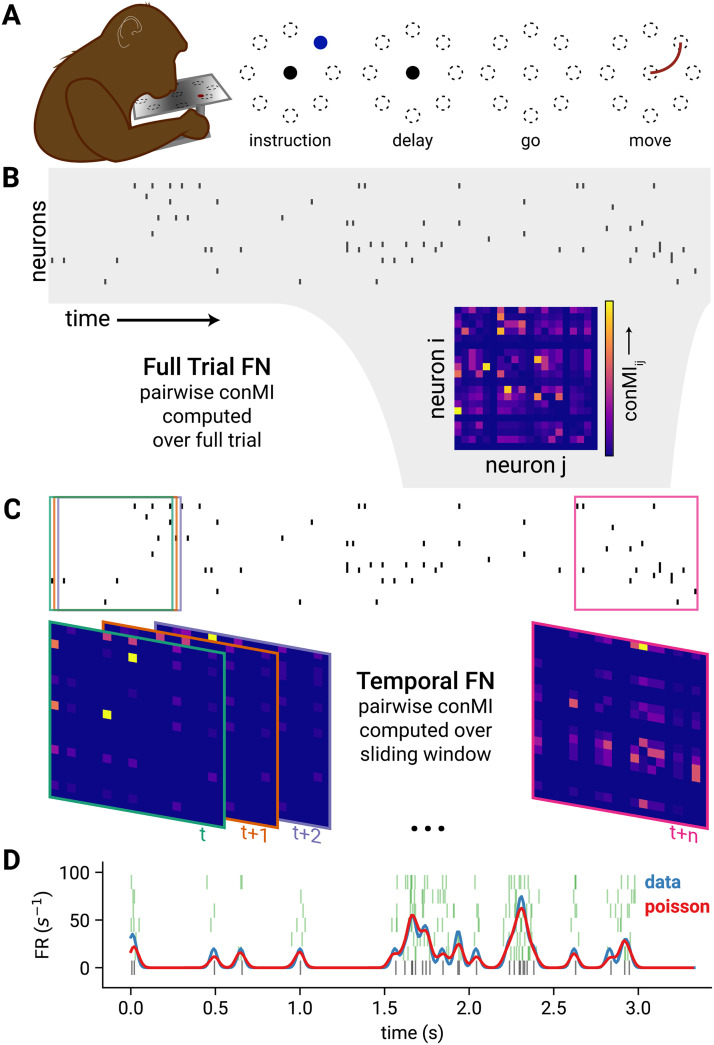
Task structure and functional network construction. (A) Subject using a Kinarm exoskeleton to control a cursor on a screen. The subject holds the cursor at the center target (black circle) to initiate a trial. An *Instruction* cue (blue) appears in one of eight target locations (indicated by the circles with broken lines). After the delay period the target cue begins blinking, which indicates that the subject may move to the instructed target. (B) A raster plot shows the binned spikes of the recorded neurons across time. The full trial FN is constructed by computing the confluent mutual information (conMI; see Methods) between the spike train for the full trial for each pair of recorded neurons. (C) To generate the temporal FN, pairwise conMI were computed within a 200-ms sliding window across the trial. Boxes on the raster indicate the interval in the spike train that was considered for the corresponding FN with the same color border. (D) To generate rate-matched nulls, we computed the instantaneous firing rate of a unit for each single trial (blue trace) from the raw spikes (black raster plot, bottom). We used this firing rate profile to generate new spikes from an inhomogeneous Poisson process (five example spike trains, green rasters). The mean of the instantaneous firing rates for 50 iterations of this Poisson process is shown in red.

We analyzed single-unit recordings from M1 while two macaques (Monkey Rs and Monkey Rj) performed a center-out reaching task in the horizontal plane. Subjects were trained to control a cursor on a screen by moving a handle on a 2D arm exoskeleton (Kinarm, Kingston, Ontario). The task was to hold the cursor in the center position during an instructed-delay period while an *Instruction* cue was presented that indicated one of eight target reach locations positioned radially around the center. After the end of the delay period (1 s), a *Go* cue appeared instructing the subjects to initiate a reaching movement (Figure 1A).

We summarized neuronal population activity during the task as a functional network (FN, Figure 1B–C). The FN is composed of nodes, which are the spiking single units, and edges between nodes, which correspond to the pairwise spike time statistics and not anatomical connectivity (Rs: 142 single units, Rj: 78 single units). Specifically, edges are computed using a mutual information measure (Chambers et al., 2018). Confluent mutual information (conMI; see the Methods section) computes the mutual information between the binned activity of neuron *i* at time *t*, and of neuron *j* at time *t* and the adjacent time bin, *t* + 1 (where bins are 10 ms), and it is positive, by definition. The reliability of the coactivity between neuron *i* and *j* during a specified time window resulted in a weight. The resultant FN is both weighted and directed corresponding to the reliability and the directionality of the pairwise spike time correlation between units. We assessed whether the conMI between *i* and *j* is strongly influenced by their firing rates (FR). We found that the relationship between conMI and pairwise FR (geometric mean) is not a simple linear correlation (see Supplementary Figure 1 in the Supporting Information). Notably, the relationship between conMI and the FR is similar in both the data and the rate-matched null.

### Correlational Structure in Population Activity Is Specific to Reach Direction

**Figure 2.**
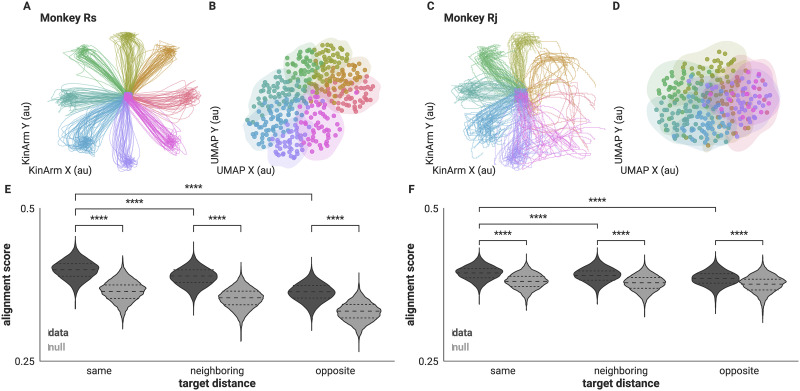
Correlational structure of full trial FNs are specific to reach target direction. (A, C) Hand trajectories of included trials colored by reach direction. (B, D) Low-dimensional embedding of full trial FNs preserve reach target direction. Colored the same as panels A, C. Shaded contour is the bivariate kernel density estimation (see Methods) for the (x, y) positions of the FNs to visualize the boundaries of the projected FNs of each direction. (E, F) Distribution of alignment scores between each pair of FNs separated by target distance in dark gray and score distributions for corresponding rate-matched FNs shown in light gray (**** *p* < 1.00e−04, MWU two-sided test). Dashed lines indicate quartiles.

We first determined whether FNs during the entirety of single reach trials are specific to reach target direction. We did so in two ways: low-dimensional embedding of FNs and a graph similarity metric. Both approaches showed that FNs constructed from closer reach target directions are also closer in network space. First, we embedded the FNs in a low-dimensional manifold that optimizes the distances between data points according to the original high-dimensional similarity. Consequently, FNs that are more similar to each other will lie closer together on the low-dimensional manifold, and conversely, FNs that are structurally different will be more distant. Specifically, we used UMAP to project the high-dimensional FNs into a lower dimensional space (see Methods). We found that networks from reaches to neighboring directions are close together in the low-dimensional manifold. In fact, the embedding produced a radial arrangement of data points that reflected the radial arrangement of the target locations (Figure 2A–D).

To further evaluate the similarities and differences between FNs according to reach direction as well as control for similarities and differences resulting from firing rates, we compared FNs and single trial rate-matched FN nulls using graph alignment scores (GAS; see Methods). GAS allows for a quantitative comparison of FNs by identifying common edges between graphs (Gemmetto et al., 2016). This metric preserves node identities and reflects the fraction of similar edges out of all the existing edges between two networks. Therefore, a value of 1 means that the two networks are exactly the same. We measured alignment between each pair of FNs constructed from single trials. We then grouped the measured GAS according to the degree difference between the respective instructed reach targets: same (Δ0°), neighboring (Δ45°), and opposite (Δ180°). We observe that pairs of FNs from trials with the same reach target have higher alignment scores (Δ0° Rs = 0.399 ± 0.014, Rj = 0.393 ± 0.011, mean ± *SD*) than those from neighboring reach directions (Δ45°: Rs = 0.388 ± 0.015, Rj = 0.389 ± 0.011; *p* ≤ 1.865e−89, Mann-Whitney U (MWU) two-sided test, mean ± *SD*). Alignment scores were lowest when computed using FNs from opposite reach targets (Δ180°: Rs = 0.363 ± 0.015, Rj = 0.385 ± 0.012; *p* ≤ 1.882e−236, MWU two-sided test, mean ± *SD*), indicating that the shared correlational network structure, in this case summarized as FNs, are informative of the instructed reach (Figure 2E–F). Additionally, while the rate-matched networks also exhibited differences in these distributions, GAS from the data were higher for all three distributions, suggesting that FNs are more consistent than firing rates trial to trial (Rs = 0.363 ± 0.016, 0.353 ± 0.016, 0.331 ± 0.016; Rj = 0.380 ± 0.012, 0.378 ± 0.012, 0.374 ± 0.014; GAS for pairs of rate-matched FNs for same, neighboring, and opposite trials, respectively, *p* < 0.001, MWU two-sided test for all data and rate-matched comparisons, mean ± *SD*). Together both measures, UMAP and GAS, demonstrated that there were differences in the pairwise spike time correlation structure within the recorded population across different reach directions, and that there was additional structure resulting from pairwise spike time statistics of the populations that was not accounted for by firing rate correlations.

### Temporal Progression of Pairwise Spike Time Statistics on Single Trials Are Specific to Reach Direction

**Figure 3.**
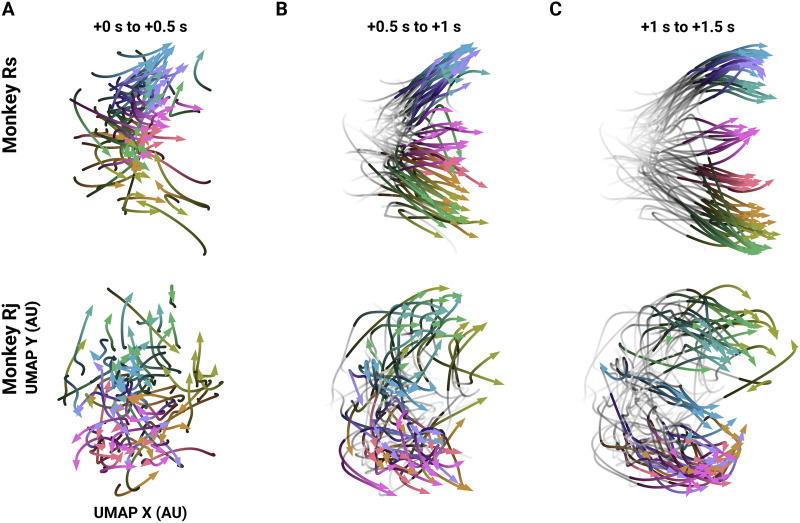
Trajectories of single trial temporal FNs through a low-dimensional subspace. Example trajectories of single trial temporal FNs across a low-dimensional subspace (10 trials per direction). Times (in seconds) indicate the time post-instruction. (A) Trajectories from instruction (0 s) to 0.5 s after instruction. Trajectory begins with the darkest hue. Color legend is the same as Figure 2A–D. (B) The same trajectories from panel A later in time: the gray, low-opacity tails show the trajectory from *Instruction*, and the colored and opaque section shows the trajectory at 0.5 s post-instruction to *Go* cue (1 s post-instruction). (C) The same as panel B but for after *Go* (1 s) and through the end of the trial.

Correlations between neurons are dynamic (Aertsen et al., 1989) and can vary over a behavioral trial depending on task condition (Vaadia et al., 1995). Thus, we next evaluated the time-varying spike time correlational structure by constructing FNs from short epochs (200 ms) of trial time. These “snapshots” of the network across time, *temporal FNs*, allowed us to evaluate how the network evolves over the time course of single trials and to determine when, during a trial, the reach-specific differences in the FNs first occur and are most pronounced. Again, we examined the differences between FNs in two ways. First, we embedded the FNs into a lower dimensional subspace using UMAP, as we had done with the full trial FNs. This allowed us to use the embedding to track the progression of temporal FNs relative to trial structure and reach direction. We found that the temporal FNs progressed along trajectories through the subspace in a reach-specific path (Figure 3). Temporal FNs are initially close together in the computed subspace (Figure 3A) and then increasingly separate according to the instructed reach direction as the trial progresses (Figure 3B), separating maximally after the *Go* cue and during movement (Figure 3C). Again, we used an unsupervised method of dimensionality reduction and therefore did not include information about the behavior of the subject in the embedding. Consequently, any separation in the embedding of the temporal FNs is the result of the differences in the pairwise spike time statistics of the neural population and not from any training labels.

**Figure 4.**
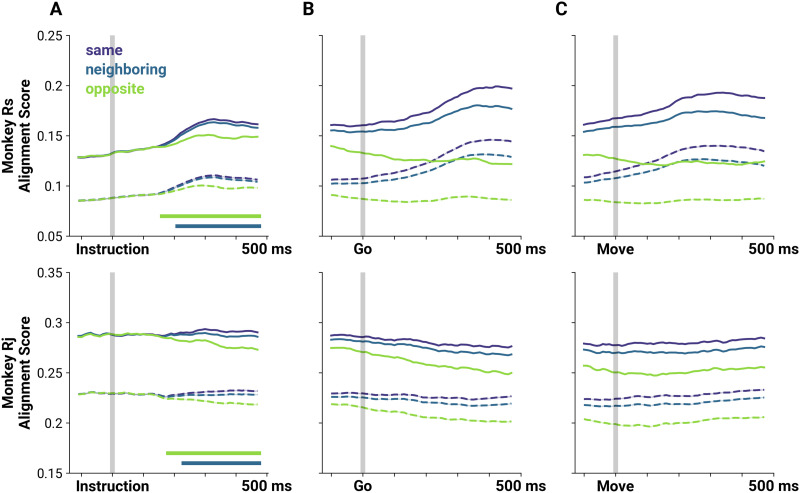
Graph alignment scores between FNs reflect distance of reach targets. (A) Mean alignment scores between pairs of FNs at each sliding window (solid lines), and corresponding scores for rate-matched FNs (broken lines) aligned to the *Instruction* cue. The shaded region indicates the standard error of the GAS distribution, which is very small. Alignment scores are separated based on reach target difference: same (violet), neighboring (blue), and opposite (green) reach targets. Straight horizontal lines on the lower left of the plot indicate when the score distributions from neighboring or opposite locations (blue and green, respectively) become significantly different from the score distributions between FNs of the same direction (*p* < 0.01, MWU two-sided test, Bonferroni corrected). (B) The same as panel A but aligned to the *Go* cue. Differences between distributions are significant throughout the plotted window (*p* < 0.01, MWU two-sided test, Bonferroni corrected). (C) The same as panel A but aligned to movement onset. Differences between distributions are significant throughout the plotted window (*p* < 0.01, MWU two-sided test, Bonferroni corrected).

As with the full trial FNs, we next computed the extent to which the structure of the temporal FNs indicated the instructed reach and compared the temporal FNs with the rate-matched FNs. At each time window, we measured the GAS of every pair of FNs and sorted the scores according to difference in target direction. We then evaluated the distribution of graph alignment scores for the same, neighboring, and opposite reach directions across the time course of the trial (Figure 4). Before and shortly after instruction, the FNs are not informative as to reach direction as indicated by similar GAS values (MWU two-sided test, *p* ≥ 4.045e−3, Bonferroni corrected between all scores from data FNs). The score distributions of neighboring and opposite reach directions became significantly different from scores of FNs from the same reach trials after the *Instruction* cue, consistent with the embedding result (Figure 4A; Δ0° vs. Δ180°: MWU two-sided test, *p* ≤ 1.576e−6, Bonferroni corrected from 160 ms post-instruction for Rs, and 180 ms post-instruction for Rj. Δ0° vs Δ45°: MWU two-sided test, *p* ≤ 3.106e−4, Bonferroni corrected from 210 ms post-instruction for Rs, and 230 s post-instruction for Rj). The scores remained significantly different for the remainder of the trial and also at movement onset (Figure 4B–C). Notably, the scores between the same and the opposite reach directions diverge first before scores from the same and neighboring reach directions, again suggesting that FNs generated from nearby reach directions are also more similar in network space. We also note that while the rate-matched FNs exhibited differences between distributions (Δ0° vs. Δ180°: MWU two-sided test, *p* ≤ 2.305e−4, Bonferroni corrected from 160 ms post-instruction for Rs and Rj. Δ0° vs Δ45°: MWU two-sided test, *p* ≤ 8.688e−4, Bonferroni corrected from 220 ms post-instruction for Rs, and 229 ms post-instruction for Rj), the GAS between pairs of FNs computed from pairwise spike time statistics from the data are consistently higher, indicating that the structure of pairwise spike timing is conserved from trial to trial. Additionally, if the temporal evolution in alignment scores are due to rates alone and if the GAS values for the data are merely offset by some value, we would expect that the normalized GAS (see Supplementary Figure 2 in the Supporting Information) would show neither (a) changes across time nor (b) significant differences in the GAS values between reach directions. We show that when we normalize the GAS we still find that for Rs, GAS between Δ0 and Δ45 are significantly different at 210 ms (similar to the non-normalized GAS). For Rj, while the mean normalized GAS for Δ0 is consistently higher than the mean for Δ45 starting at 110 ms, the scores are significantly different at 380 ms. However, it must also be noted that the scores were significantly different between Δ0 and Δ90 starting at 220 ms for Rj.

### Decoding Behavior From Functional Networks

**Figure 5.**
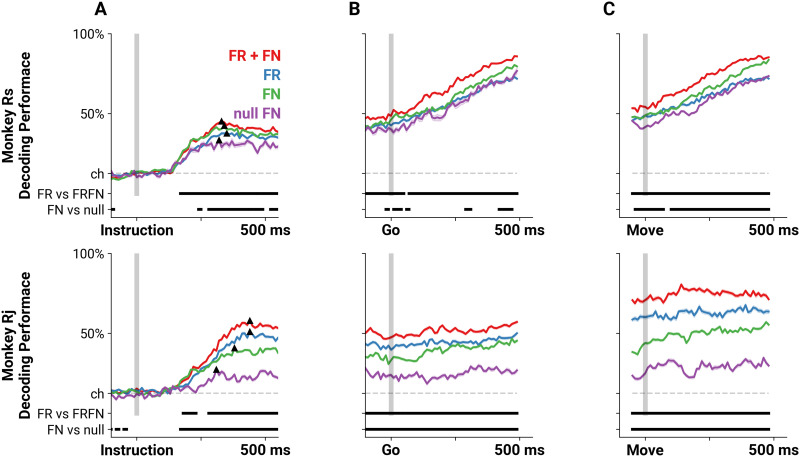
Decoders that incorporate pairwise spike time statistics predict reach direction more accurately. Mean performance perceptron decoders trained on either firing rates (FR, blue), the set of pairwise correlations as FNs (FN, green), both firing rates and correlations (FR + FN, red), or correlations from rate-matched nulls (null FN, purple) aligned on *Instruction* cue (A), *Go* cue (B), and movement onset (C). Dashed gray line indicates chance level (one in eight), and the two bars on the bottom of each panel indicate the times during which performance of the FR and FRFN, or the FN and null FN decoders are significantly different from each other (*p* < 0.01, MWU two-sided test, Bonferroni corrected). The triangles in panel A mark the initial peak in performance for the decoders. The shading represents the standard error of the decoder performance.

Temporal FNs became increasingly differentiable during the trial with the differences following a stereotyped time course relative to trial structure, indicating that there is information about motor behavior in the temporal FN. We next asked whether this information can be used to decode reach target direction. Since GAS analysis indicated that the differences in the temporal FN depended on time, we also measured when the temporal FNs were decodable relative to trial structure and movement onset, and when they were maximally decodable. To do so, we employed multilayer perceptron decoders trained on different features of the data to predict the reach target of a 200-ms sample window of a single trial (see Methods). We then compared the performance of the decoders based on the features on which they were trained. Specifically, decoders were trained on either the set of pairwise spike time statistics between neurons generated in the time window (FN decoder, Figure 5, green), or the firing rates of the neurons during the window (FR decoder, Figure 5, blue). To determine whether pairwise spike time statistics carry additional information beyond firing rates, we also examined decoding performance when both firing rate and the temporal FNs were included in decoder training (FRFN decoder, Figure 5, red). Comparing the decoding performance of the FN and FR decoders to the full FRFN decoder allowed us to establish the contribution of each held-out feature to the overall decoding performance. Finally, in order to rule out the effect of spurious correlations due to firing rate dynamics, we generated FNs from trial-by-trial Poisson rate-matched neurons and similarly evaluated decoding performance (null FN decoder, Figure 5, purple; see Methods).

As expected, before the *Instruction* cue, all four decoders performed at chance level (Figure 5A, 12.5% performance indicated by dashed line). At 147 ± 9 ms following the *Instruction* cue, decoding performance increased above chance for the FN, FR, and FRFN decoders (one-sample *t* test, *p* ≤ 9.550e−4, Bonferroni corrected, mean ± *SD*). The null FN decoders achieved performance above chance about 50 ms later, at 205 ± 44 ms (mean ± *SD*). All four decoders reached an initial peak around 364 ± 48 ms (defined as the maximum performance within 500 ms of the instruction, indicated by the black triangle on every performance trace in Figure 5). At this peak, the FRFN decoders performed better than the FR decoder (Rs: 45.10% vs. 37.67%, Rj: 58.03% vs. 51.03%, respectively, *p* ≤ 6.438e−05, MWU two-sided test), suggesting that the inclusion of pairwise spike time statistics provides additional information about the reach direction. In fact, the FRFN decoder performance was significantly higher than the performance of the FR decoder beginning at 170 ms for Rs and 180 ms for Rj and remained significantly higher throughout the rest of the trial, as well as during movement (MWU two-sided test, *p* ≤ 9.888e−04, Bonferroni corrected between FRFN and FR performance scores). We note that the FR decoder performed better than the FN decoder for Rj (51.03% vs. 40.85% at their initial peaks, respectively). However, the increase in performance in the combined FRFN decoder compared with the FR decoder indicates that precise spike time correlations contain additional information about the reach target beyond firing rates alone.

We also observed that at the initial peak, the FN decoders performed significantly better than the rate-matched FNs (Rs: 42.39% vs. 33.37%, Rj: 40.85% vs. 27.28%, respectively, *p* ≤ 3.562e−04, MWU two-sided test). For Rj, this performance difference was significant from 170 ms throughout the rest of the trial (MWU two-sided test, *p* ≤ 3.3845e−04, Bonferroni corrected). For Rs, this difference was significant beginning at 240 ms (MWU two-sided test, *p* ≤ 8.853e−04, Bonferroni corrected) and sporadically thereafter. This highlights that there is information about the instructed reach in the pairwise spike time statistics in the data that does not arise from chance correlations due to firing rates.

### Reciprocity in the Network Varies Systematically During the Trial

**Figure 6.**
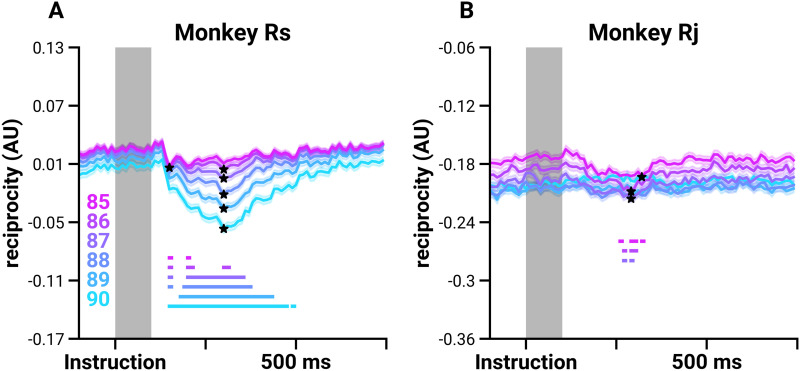
Reciprocity decreases shortly after instruction. Mean normalized reciprocity scores (lines; the shaded region shows the standard error) from thresholded FNs based on weight percentile values for Monkey Rs (A) and Monkey Rj (B) indicated by the color legend in the lower left. The mean reciprocity for the shaded region (*Instruction* to 100 ms post-instruction) is computed for each trial, resulting in a distribution of reciprocity scores, which is then compared with the scores at each time point and at the minima to determine whether there is a decrease in reciprocity. Stars indicate whether the distributions at the minima decrease significantly (*p* < 0.01, independent sample *t* test). Solid straight lines at the bottom of the plot show the times that are significantly different from the baseline (*p* < 0.01, independent sample *t* test, Bonferroni corrected).

One of the strengths of representing cortical population activity as a weighted and directed temporal FN is that not only can we investigate the dynamics of pairwise spike time statistics across the population (as weighted edges), we can also consider the changes to network architecture. Doing so has the potential to provide insights into information propagation and computation. Here, we focus on the most reliable reciprocal connections in the temporal FN. We thresholded the temporal FNs according to weight percentile, in order to isolate the most reliable edges, and then measured the normalized reciprocity of the temporal FNs throughout the trial (see Methods). Reciprocity tells us how likely two units in the temporal FN are mutually linked (Squartini et al., 2013) as well as the importance of the direction of the interaction between neurons: A fully reciprocal FN means that units are coactive symmetrically, in contrast to a less reciprocal FN where the direction of the interaction is important in characterizing the network. In the temporal regime, reciprocity may influence the state of the FNs in the next time point. We investigated whether and when the reciprocity of the temporal FN changes over the time course of the trial. We compared the mean scores of each trial from instruction to 100 ms post-instruction (Figure 6, gray shaded region; defined as the baseline, see Methods) with the mean scores at each time point during the delay period. Reciprocity significantly decreases at 156 ± 11.1 ms for Rs and 267 ± 4.7 ms for Rj (independent sample *t* test, *p* ≤ 7.46e−03 and *p* ≤ 9.83e−03, respectively). The significant decrease in reciprocity lasts until 358 ± 91.4 ms for Rs and 303 ± 12.5 ms for Rj.

We also compared the reciprocity scores at the time when the mean reciprocity over trials is at its minimum (Figure 6, indicated by ★) with the mean of the baseline distribution. The minima occurred at 291 ± 31 ms after the *Instruction* cue (mean ± *SD*). We found that reciprocity is significantly decreased at the minima for the networks thresholded at 85th to 87th percentile for both Rs and Rj (Figure 6, independent sample *t* test, *p* ≤ 3.773e−4). There is a significant decrease in reciprocity with Rs at the higher threshold levels (88th to 90th percentile of edge weights), and a significant decrease in reciprocity scores with Rj at lower thresholds (75th to 85th percentiles; see Supplementary Figure 3 in the Supporting Information). The difference in significant threshold levels is due to the differences in the density and size of the FNs. Nevertheless, this decrease is present in both subjects. At moments when inputs external to the recorded population would be most likely, the network structure becomes less reciprocal.

## DISCUSSION

We show that pairwise spike time statistics between neurons, summarized as functional networks (FNs), are informative of reach direction and are dynamic over the course of a single task trial. Previous studies have shown that firing rate correlations between neurons provide additional information about behavior or the environment that is not gleaned from observing each neuron independently (Maynard et al., 1999). Moreover, the dynamics of pairwise firing rate correlations are indicative of behavioral conditions that are otherwise unobservable from individual neuron firing rates or from trial-averaged cross-correlations (Vaadia et al., 1995). These previous studies measured correlations of spike counts across trials and over broad timescales. In contrast we focused on spike time correlations over short intervals. Earlier work has documented fine timescale synchrony between pairs of M1 neurons that emerges predominantly at movement onset and carries information about reach direction (Hatsopoulos et al., 1998). Our results are consistent with this earlier work but extends it in several ways. First and foremost, our focus here was on characterizing the FN structure among the entire recorded neuronal population as opposed to multiple, individual pairwise interactions. Second, by using conMI, we could distinguish directed functional interactions as opposed to synchronous interactions. This allowed us to analyze the temporal dynamics of reciprocal versus nonreciprocal interactions across the duration of the reach. Third, we documented the emergence of information-bearing interactions early during movement preparation in the instructed-delay period, which was not evident in the earlier work.

Since movement is the culmination of multiple computations, many of which happen at short timescales, we hypothesized that task-relevant information must be represented in the short timescale pairwise spike time statistics. Our work substantiates this hypothesis, since we find evidence that temporal FNs constructed from short intervals during single trials carry a representation of the instructed movement. Specifically, we found that the similarity between pairs of temporal FNs from single trials reflected the distance between their respective target directions and are correspondingly decodable. The temporal FN framework also allows us to identify *when* correlations begin to be indicative of reach direction, which in turn has the potential to provide insight into corresponding computations. We find that FNs become decodable above chance 140 ± 10 ms after the *Instruction* cue appeared. Notably, an increase in timing precision of spiking in individual neurons across trials has been found around this time (∼100 ms), and mutual information increases between target location and spike counts of single neurons at this time (Reimer & Hatsopoulos, 2010). The modulation of firing rates across the population and the stronger relationship between spike count and target location suggest that information about the target arrives at M1 at this time lag. Consistently, we find that precise spike timing *between* neurons in single trials, as reflected in their correlation, is additionally informative of target direction. Moreover, alignment scores between temporal FNs constructed from single trials of the same target direction are higher than alignment between temporal FNs that correspond to neighboring and opposite directions, suggesting that there is consistent and informative spike timing between neurons on a trial-to-trial basis that is specific to reach direction.

Local connectivity patterns shape the activity patterns and consequently, the dimensionality and behavior of a recurrent network (Recanatesi et al., 2019). In this work, we focused on measuring reciprocal connections because they serve as building blocks for higher order (beyond pairwise) structures. When external inputs impinge on local circuitry, they must be integrated into ongoing activity and processed across the network. A recent study that inferred single trial M1 dynamics using LFADS (latent factor analysis via dynamical systems) found that it was necessary to include external inputs in order to maximize model accuracy (Malonis et al., 2021). They found that large input transients were disproportionately necessary at ∼150 ms after target presentation compared with other times in the trial. Here we find that reciprocal connections begin to decrease with similar latencies to the target appearance as the inferred input transients (significantly at 156 ± 11.1 ms for Rs and 267 ± 4.7 ms for Rj). Also at this time, decoding performance improves above chance (147 ± 9 ms), and GAS values between the same and opposite directions begin to diverge (170 ± 10 ms). This shift in structure may be an indication of the network integrating and propagating information about inputs external to the recorded population, such as a visual stimulus related to the instructed target direction. Interestingly, this topology is transient; the network returns to its baseline level of reciprocity, but it is important to note that the information about the reach target is maintained in the FN after this transient topological shift, since decodability of the FNs remains stable for the remainder of the delay period. Previous studies have shown that the population activity during the preparatory and the movement phase are dynamic (Churchland et al., 2012; Shenoy et al., 2013; Vyas et al., 2020), that they occupy orthogonal subspaces (Kaufman et al., 2014), and that these subspaces can be linked (Elsayed et al., 2016). Consistent with this work on computation through population dynamics, we show that the patterns of coordinated activity of the neural population evolves during the reach trial in a systematic way. Our findings provide evidence that the network transiently reorganizes to a less reciprocal topology that may facilitate processing external inputs, setting the state of the population according to the instructed movement, and may enable the reported transition from the preparatory subspace to the movement subspace.

In this study we did not try to classify the single units functionally or into putative cell classes. Future work would clearly benefit from data in which individual classes of neurons are considered separately. The FN framework can be extended to include labels on nodes and edges such as cell type, or functional properties (Faskowitz et al., 2022). Notably, an impressive census of cell types in motor cortex has recently been published by the BRAIN Initiative Cell Census Network (BICCN, 2021) highlighting the potential for an analytical approach, such as FNs, that can incorporate both cell information and network dynamics. For example, in the visual cortex the topology of single trial FNs depends on the visual stimulus, and untuned neurons act as hubs in the network, serving an integral role in stimulus coding (M. Levy et al., 2020). A functional network framework revealed that frontoparietal areas in the grasping motor network have modular organization that is not constrained to anatomical boundaries, and a rich club of oscillatory neurons that may be key to synchronizing the network and generating grasping behavior (Dann et al., 2016). Previous work has shown that modeling functional connections can predict neural responses in M1 more accurately than canonical tuning curves, suggesting that the tuning properties of individual neurons is in part a result of network interactions (Stevenson et al., 2012). Additionally, tuning properties are not stationary such that single cortical neurons in M1 encode temporally extensive movement fragments or trajectories, as opposed to single movement parameters such as direction (Hatsopoulos et al., 2007). The temporal FN framework that we introduce in this paper has the potential to link dynamically changing network interactions across the trial to time-varying preferred movement trajectory tuning.

Aside from containing information about the instructed reach, upon visual inspection of the low-dimensional embeddings, we found that FNs carry kinematic information. For example, in the full trial and temporal embeddings of FNs from Rj (Figure 2C–D, Figure 3, bottom row), overlapping projected FNs show trials that had similar kinematic trajectories despite different target directions. Rj’s kinematic trajectories towards the lower right targets were not straight towards the target (pink in Figure 2C and Figure 3 bottom row); Rj moves straight down and then right. We found that FN embeddings where the target is the lower right (pink) and the one straight down (violet) are correspondingly overlapping. Additionally, low-dimensional embeddings of temporal FNs separate into two main branches: temporal paths of the FNs for reach directions to the lower left targets (hand trajectories shown in Figure 2A and C) tended to be closer together, forming a “branch” of paths, and the paths for the upper right targets form another. Specifically, in Rs (Figure 3, top row), the upper branch of paths corresponds to pulls towards the body. Conversely, the lower branch corresponds to reaches that require the subject to push away from the body. This suggests that, while we have performed these analyses on data where the subject is performing an eight-direction center-out task, FNs may be able to capture a wider range of movement directions and kinematic variability than explored here.

FNs are a useful tool to achieve insight into M1 network dynamics that correspond to motor behavior. FNs provide a tractable summary of population activity that is decodable and can provide insight into how the state of the system changes over the time course of a trial. The neural interactions summarized by FNs influence spiking activity of the population that can be, in turn, read out by downstream agents (M. Levy et al., 2020). Our results suggest that FNs reorganize according to motor goals and argue that a functional network examination of motor cortical FNs may provide insights into the underlying circuit mechanisms that drive these cortical population responses and ultimately motor behavior.

## METHODS

### Data Collection and Reaching Task

We used previously published datasets from two macaques, Monkey Rs and Monkey Rj, performing an instructed center-out reaching task (Hatsopoulos et al., 2004; O’Leary & Hatsopoulos, 2006). Subjects were trained to hold a cursor on a center target presented on a video screen using a 2D arm exoskeleton (Kinarm, Kingston, Ontario). One of eight radially positioned peripheral targets was then presented and served as an *Instruction* cue during which time the subjects were required to keep holding the cursor on the center target. After a 1-s delay period, the peripheral target began blinking (*Go* cue), instructing the subjects to move the cursor to the peripheral target (Figure 1A). Trial start was 0.5 s before the *Instruction* cue appeared, and trial termination was 0.5 s after the peripheral target was acquired. Trial inclusion depended upon target acquisition falling within 1.5 s following movement onset. We also included only correct trials. Movement onset is defined as the time when the hand velocity reached 5% of the peak velocity of the movement after the *Go* cue.

Neural data were recorded from 96-channel Utah arrays implanted in the arm/hand area of primary motor cortex (M1) on the precentral gyrus. Spike waveform snippets sampled at 30 kHz were extracted using a user-defined threshold (Cerebus Blackrock Microsystems, Salt Lake City, UT) and were sorted into individual units using Offline Sorter (Plexon, Dallas, TX).

The surgical and behavioral procedures involved in this study were approved by the University of Chicago Institutional Animal Care and Use Committee.

### Using Pairwise Spike Time Statistics to Construct Functional Networks

To compute pairwise spike time statistics between recorded neurons, we binned the recorded spike trains into 10-ms bins, assigning a value of 1 if at least one spike occurred in that bin, and 0 otherwise. We then used the confluent mutual information (conMI) between the binned spike train (Chambers et al., 2018). ConMI tells us how much information we gain from the firing state of a source neuron *i* at time *t* about the firing state of target neuron *j* in the same time bin *t* and the consecutive bin, *t* + 1:

$$\text{conMI}=\sum_{i\left(t\right)\in \left\{0,1\right\}}\sum_{j\left(\hat{t}\right)\in \left\{0,1\right\}}p\left(i\left(t\right),j\left(\hat{t}\right)\right)\cdot {\text{log}}_{2}\left[\frac{p\left(i\left(t\right),j\left(\hat{t}\right)\right)}{p\left(i\left(t\right)\right)\cdot p\left(j\left(\hat{t}\right)\right)}\right],$$

$$\text{where}\;j\left(\hat{t}\right)=\left\{\begin{array}{cc} 1, & \text{if}\;j\left(t\right)=1\;\text{OR}\;j\left(t+1\right)=1\\ 0, & \text{otherwise} \end{array}\right..$$

The functional network constructed using this measure is consequently weighted and directed. We computed functional networks from either the full trial duration or from 200-ms epochs. For the full trial networks, we computed the conMI between the entire spike train of the source and target neurons for a single trial (Figure 1B). We also computed the conMI for 200-ms windows that we slid in 10-ms (1-bin) increments across a single trial, resulting in a temporal FN per trial: a set of FNs each representing the measured coactivity of the recorded population during the trial at each time window (Figure 1C).

To disambiguate the structure that arises from firing rates alone and that which is the consequence of precise spike timing, we computed FNs from short interval rate-matched Poisson neurons. Specifically, we computed the instantaneous firing rate of each neuron on a trial-by-trial basis by convolving each neurons’ spike train (Figure 1D, black raster) with a Gaussian kernel (*σ* = 20 ms). This smoothed rate-signal (Figure 1D, blue trace) is then used as the probability of observing a spike for that neuron within a sampling period (10 ms) in an inhomogeneous Poisson process (Elephant RRID:SCR_003833, Denker 2018), which results in a spike train with the same rate profile over the time course of a trial as the original data, but the precise timing, which is crucial to compute pairwise spike time statistics, is jittered within the width of the Gaussian kernel (Figure 1D, green rasters and mean instantaneous rate in red). We generate a rate-matched spike train for each neuron for every trial, and then generate the rate-matched FNs using the methods described above.

### Dimensionality Reduction and Visualization

We vectorized each FN adjacency matrix and used the UMAP algorithm to reduce the dimensionality of the FN adjacency matrix from *N*-by-*N* (neurons, *N*: Rs: *N* = 143, and Rj: *N* = 78) to two dimensions (McInnes et al., 2018). For the full trial FNs we used the following UMAP parameters: n_neighbors = 50, min_dist = 1, metric = 'cosine'. Each sample used to construct the low-dimensional embedding is a FN computed from an entire trial (total samples: Rs = 391, Rj = 246). We used a bivariate kernel density estimation for the (x, y) positions of the FNs to visualize the boundaries of the projected FNs for each reach direction. As with the full trial FN, we embedded all the temporal FNs together, but in this case each sample is a FN computed from 200 ms in the trial (total samples: Rs = 103,246, Rj = 20,856). For the temporal FNs, because there are significantly more samples, we used a different set of parameters in order to balance the local versus global structure of the data accordingly (n_neighbors = 100, min_dist = 0.1, metric = 'cosine'). We then used a spline to interpolate a path through the set of embedded FNs from a single trial to visualize the trajectory of the temporal FN in the low-dimensional subspace.

### Graph Alignment Score

Similarity between two FNs, M and N with *k* neurons, was measured using a node-identity preserving graph alignment score (GAS), as described in M. Levy et al. (2020) and Gemmetto et al. (2016):

$$\text{GAS}=\frac{2\sum_{\text{i}=1}^{\text{k}}\sum_{\text{j}=1}^{\text{k}}\min\left({\text{M}}_{\text{ij}},{\text{N}}_{\text{ij}}\right)}{\sum_{\text{i}=1}^{\text{k}}\sum_{\text{j}=1}^{\text{k}}{\text{M}}_{\text{ij}}+{\text{N}}_{\text{ij}}}.$$

GAS measures the ratio of overlapping edges over the total number of edges. In the weighted case, the numerator represents the sum of the minimum edge weight between each pair of nodes, and the denominator is the total sum of the edge weights. Alignment scores were grouped according to the degree difference between the instructed target in a trial. Specifically, we evaluated the GAS distributions for FNs from the same (Δ0°), neighboring (Δ45°), and opposite (Δ180°) reach directions. For the temporal networks, we computed the alignment scores between pairs of FNs within the same 200-ms time window aligned to the trial cues (*Instruction* and *Go*), and to movement onset.

### Decoding

A multilayer perceptron classifier (MLPC) was trained to decode the trial target direction from either the FN, the firing rates, both the FNs and firing rates, or the rate-matched Poisson FNs. The MLPC architecture has one hidden layer with 100 rectified linear activation units, linear output units, and a constant learning rate (step size = 0.001). The MLPC was trained for 200 iterations or until convergence, whichever happened first. We trained a different decoder for each time point and feature set (75% of the data are used for training), and tested on held-out data (the remaining 25%). We trained and tested 50 decoders for each feature set in order to get a distribution of performance scores.

### Reciprocity

We computed the weighted reciprocity of FNs using a method described in Squartini et al. (2013). First, we used a range of percentiles, from 85th to 90th (i.e., the top 15% to 10% of magnitudes), to threshold the FNs in order to isolate the most reliable interactions in the population. In order to measure the overlapping or reciprocal edges, we take the minimum weight between neuron *i* and *j*:

$$w_{\overset{⃡}{i,j}}\equiv \min\left[w_{\text{i}},w_{\text{j}}\right]=w_{\overset{⃡}{i,j}}.$$

The reciprocity of a network is the ratio between the sum of the reciprocal edge weights and the total sum of edge weights:

$$\overset{⃡}{W}\equiv \sum_{i}\sum_{j\neq i}w_{\overset{⃡}{i,j}},$$

$$W\equiv \sum_{i}\sum_{j\neq i}w_{i,j},$$

$$r\equiv \frac{\overset{⃡}{W}}{W}.$$

We normalized the value to the average reciprocity of 45 rate-matched networks ($\bar{r}$) using the equation described in Garlaschelli and Loffredo (2004) for binary networks and adapted by Squartini et al. (2013) for weighted networks:

$$\rho =\frac{r-\bar{r}}{1-\bar{r}}.$$

Thus when the value is positive, the FNs from the data are more reciprocal than what is expected from chance correlations due to firing rates, and negative when they are less reciprocal. The mean reciprocity was computed between the presentation of the *Instruction* cue to 100 ms post-instruction for every trial. This resulted in a distribution of scores that we defined as the baseline distribution. We compared the distribution of reciprocity scores at the time when the mean reciprocity over trials is at its minimum to determine whether the reciprocity of the FNs significantly changed during the trial relative to the baseline.

## ACKNOWLEDGMENTS

We thank Jacob Reimer, Zach Haga, and Dawn Paulsen for collecting the data presented in this work. We thank Mayaan Levy, Caleb Sponheim, Wei Liang, and Gabriella Wheeler Fox for their helpful comments on the manuscript.

## DATA AND CODE AVAILABILITY

The data used in this work can be found publicly at https://doi.org/10.5061/dryad.9p8cz8wm6 (Sundiang et al., 2022). All code written in support of this publication is publicly available at https://github.com/hatsopoulos-lab/macaque-dynamic_functional_networks (Hatsopoulos, 2022).

## SUPPORTING INFORMATION

Supporting information for this article is available at https://doi.org/10.1162/netn_a_00298.

## AUTHOR CONTRIBUTIONS

Marina Sundiang: Conceptualization; Data curation; Formal analysis; Investigation; Methodology; Software; Validation; Visualization; Writing – original draft; Writing – review & editing. Nicholas Hatsopoulos: Conceptualization; Data curation; Funding acquisition; Methodology; Project administration; Resources; Supervision; Writing – original draft; Writing – review & editing. Jason Neil MacLean: Conceptualization; Funding acquisition; Methodology; Project administration; Resources; Supervision; Writing – original draft; Writing – review & editing.

## FUNDING INFORMATION

Nicholas G. Hatsopoulos, NIH NINDS, Award ID: R01NS111982. Nicholas G. Hatsopoulos and Jason N. MacLean, NIH NINDS, Award ID: R01NS104898.

## COMPETING INTERESTS

N. G. H. serves as a consultant for Blackrock Microsystems, Inc., the company that sells the multielectrode arrays and acquisition system used in this study.

## TECHNICAL TERMS

Confluent mutual information: The mutual information between a source neuron at time *t*, and a target neuron at time *t* and *t* + 1.
Low-dimensional embedding: A low-dimensional space that optimizes distances between elements to match the original high-dimensional data.
Graph alignment score: A metric that quantifies the fraction of similar edges between two networks.
Reciprocity: The likelihood that two nodes are mutually connected.

## Supporting Information

https://doi.org/10.5061/dryad.9p8cz8wm6

https://github.com/hatsopoulos-lab/macaque-dynamic_functional_networks
