# Supplementary material for: Dynamic structure of motor cortical neuron coactivity carries behaviorally relevant information
Source: Netw Neurosci. 2023 Jun 30;7(2):661–78. doi: 10.1162/netn_a_00298 (PMC10312288; doi:10.1162/netn_a_00298)
Supplement: Supplementary file 1 [file netn-7-2-661-s001.pdf]

SUPPLEMENTAL FIGURES

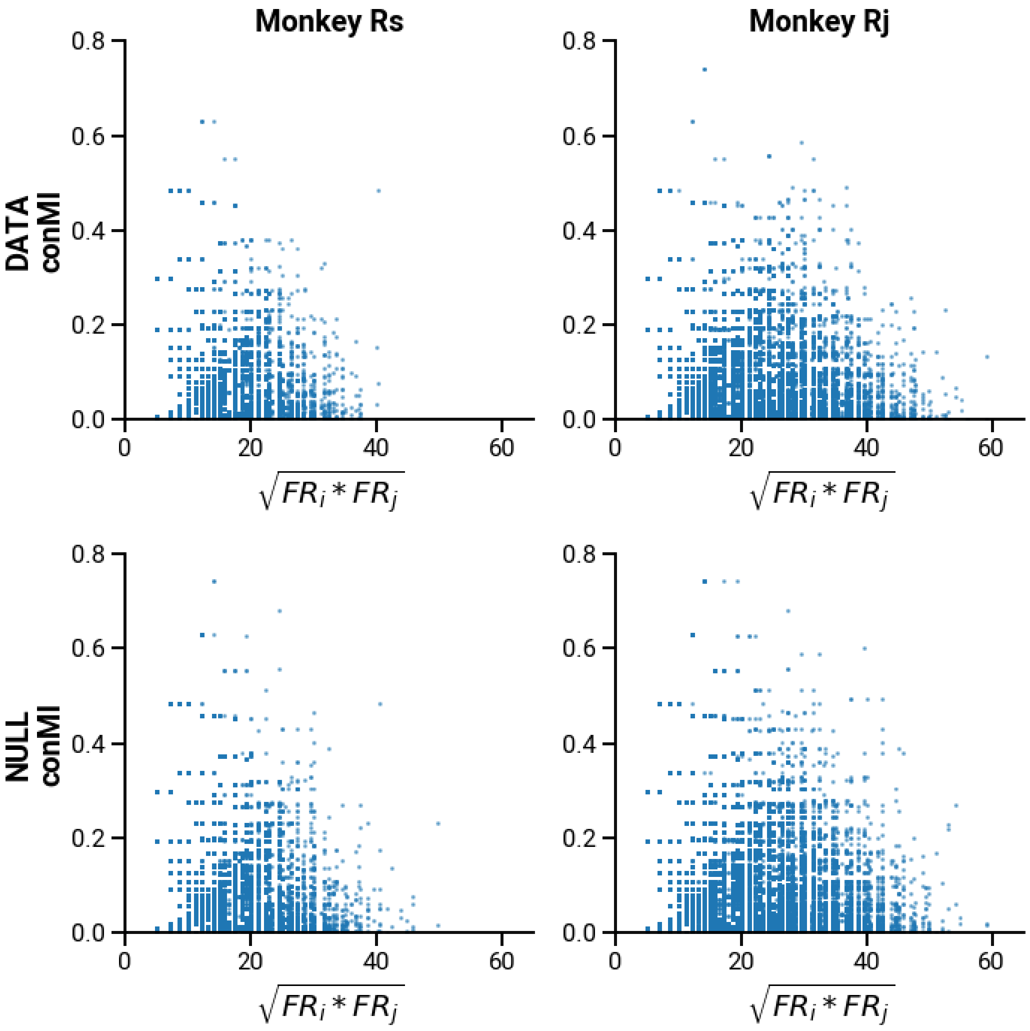

Supplementary Figure 1: Confluent Mutual Information and firing rates are related. Relationship between the geometric mean of two neurons and their measured conMI. Top row) data; Bottom row) null

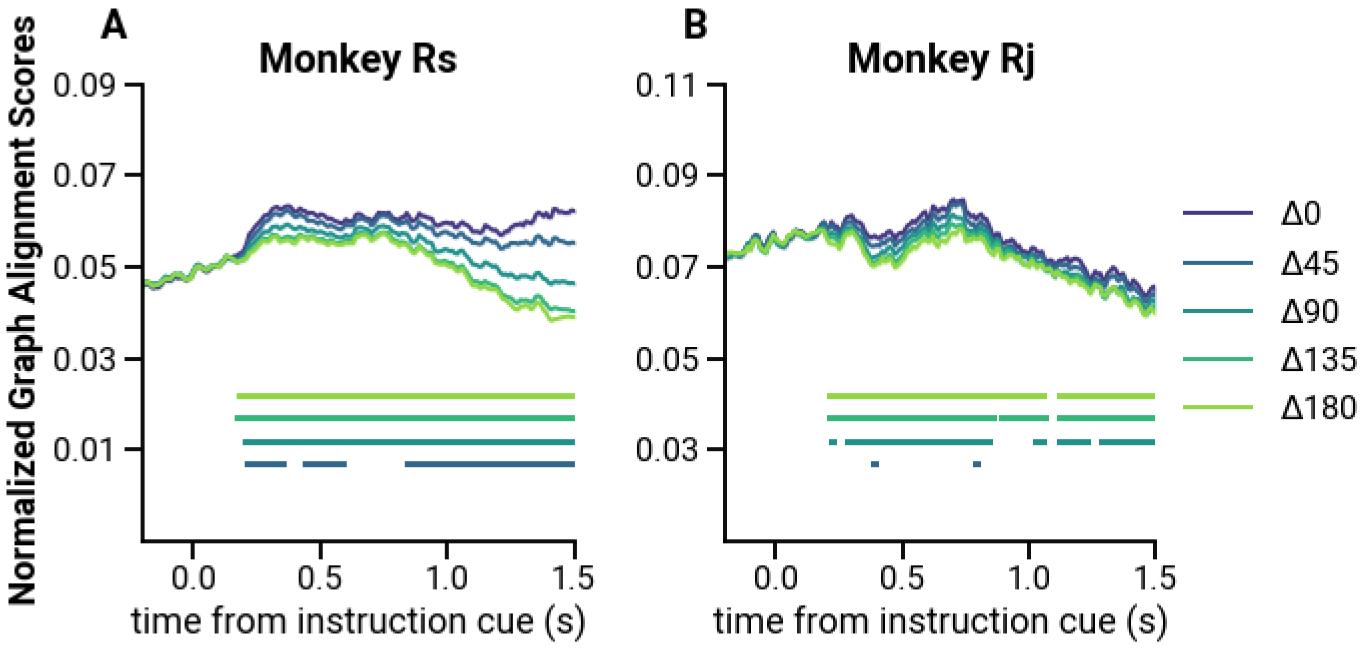

Supplementary Figure 2: Normalized Graph Alignment Scores.

Normalized GAS as described in (Gemmetto et al., 2016; Levy et al., 2020):

$$norm(GAS) = \frac{GAS - \widehat{GAS}}{1 - \widehat{GAS}}$$

Where $\widehat{GAS}$is the average GAS between two pairs of FNs for 45 rate-matched null FNs. **(A)** Mean normalized alignment scores between pairs of FNs at each sliding window aligned to the instruction cue. The shaded region, while very small, indicates the SEM of the GAS distribution. Alignment scores are separated based on reach target difference (in **Δ**degrees). Straight horizontal lines bottom plot indicate when the GAS scores from each **Δ**degree distribution become significantly different from the score distributions between FNs of the same direction (p<0.01 MWU two-sided test, Bonferroni corrected).

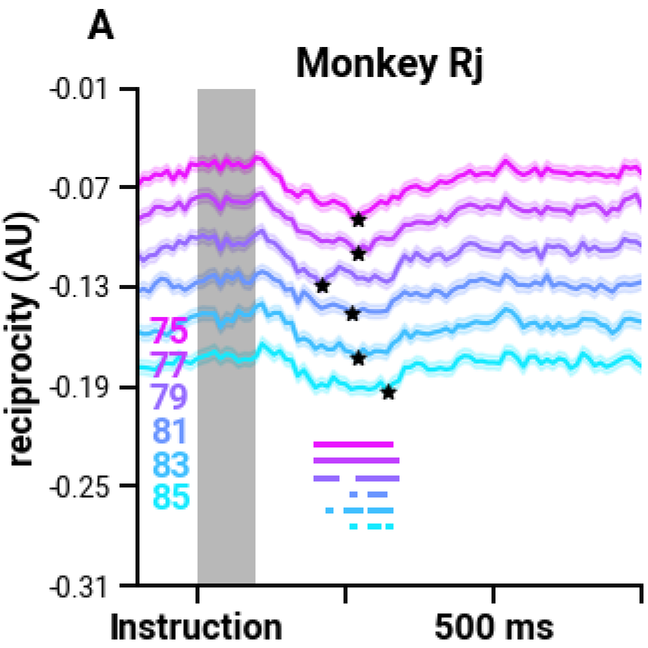

Supplementary Figure 3: Significant decrease in reciprocity in Rj between upper quartile of edge weights. Same as Fig 6 but for edge weights from the upper quartile and above.

REFERENCES CITED IN SUPPLEMENTARY FILE:

Gemmetto, V., Squartini, T., Picciolo, F., Ruzzenenti, F., & Garlaschelli, D. (2016). Multiplexity and multireciprocity in directed multiplexes. *Physical Review E*, *94*(4), 042316. https://doi.org/10.1103/PhysRevE.94.042316

Levy, M., Sporns, O., & MacLean, J. N. (2020). Network Analysis of Murine Cortical Dynamics Implicates Untuned Neurons in Visual Stimulus Coding. *Cell Reports*, *31*(2), 107483. https://doi.org/10.1016/j.celrep.2020.03.047
